# Cortical Microtubule Organization during Petal Morphogenesis in *Arabidopsis*

**DOI:** 10.3390/ijms20194913

**Published:** 2019-10-03

**Authors:** Yanqiu Yang, Weihong Huang, Endian Wu, Chentao Lin, Binqing Chen, Deshu Lin

**Affiliations:** 1 College of Life Science, Fujian Agriculture and Forestry University, Fuzhou 350002, China; yangyanqiu518@163.com (Y.Y.); 15959589920@163.com (W.H.); wedolivia@163.com (E.W.); 2 Basic Forestry and Proteomics Research Center, Fujian Agriculture and Forestry University, Fuzhou 350002, China; chentaolin@163.com

**Keywords:** Arabidopsis, petal, cell expansion, conical cell, anisotropy, cortical microtubule, microtubule organization

## Abstract

Cortical microtubules guide the direction and deposition of cellulose microfibrils to build the cell wall, which in turn influences cell expansion and plant morphogenesis. In the model plant *Arabidopsis thaliana* (Arabidopsis), petal is a relatively simple organ that contains distinct epidermal cells, such as specialized conical cells in the adaxial epidermis and relatively flat cells with several lobes in the abaxial epidermis. In the past two decades, the Arabidopsis petal has become a model experimental system for studying cell expansion and organ morphogenesis, because petals are dispensable for plant growth and reproduction. Recent advances have expanded the role of microtubule organization in modulating petal anisotropic shape formation and conical cell shaping during petal morphogenesis. Here, we summarize recent studies showing that in Arabidopsis, several genes, such as SPIKE1, Rho of plant (ROP) GTPases, and IPGA1, play critical roles in microtubule organization and cell expansion in the abaxial epidermis during petal morphogenesis. Moreover, we summarize the live-confocal imaging studies of Arabidopsis conical cells in the adaxial epidermis, which have emerged as a new cellular model. We discuss the microtubule organization pattern during conical cell shaping. Finally, we propose future directions regarding the study of petal morphogenesis and conical cell shaping.

## 1. Introduction

Plant microtubules exhibit a highly dynamic network and play pivotal roles in cell proliferation, cell expansion, and shape formation during plant development [1,2]. In interphase cells, microtubules are tethered to the plasma membrane, referred to as cortical microtubules, which serve as tracks for plasma membrane-localized cellulose synthase complexes (CSCs) and thereby guide the direction and deposition of cellulose microfibrils [3,4,5,6]. Plant cell expansion is largely defined by cell wall anisotropy, which is correlated with the orientation of cellulose microfibrils [7,8,9,10,11,12]. Therefore, the organization of microtubule arrays primarily functions in the patterning of cellulose microfibrils, which in turn contributes to cell-wall anisotropy and cell expansion. Microtubules are organized into highly dynamic arrays, which play crucial roles in the regulation of many fundamental cellular processes [13,14,15]. During plant growth and development, microtubules are often highly ordered in predominantly parallel arrays oriented perpendicularly to the axis of cell elongation.

A key question in developmental biology asks how gene activities are translated into tissue and organ growth and determination of the final shape. In flowering plants, the petal is a fascinating floral organ that differs widely with respect to color and morphology. Arabidopsis petals serve as a good experimental model system to investigate the molecular and genetic mechanisms regulating plant cell expansion and organ morphogenesis [16,17]. The petal development is largely dependent on cell proliferation during early phases of flower development, whereas the visible growth and final shape are largely controlled by post-mitotic cell expansion during the late phases of flower development [18,19,20]. Molecular and genetic studies in the past two decades have shown that a large number of regulators play critical roles in petal growth and development [21,22,23,24,25,26,27,28,29,30,31,32,33]. More than 25 years ago, microtubules have been shown to correlate with petal epidermal cell morphogenesis [34]. Despite this progress, the mechanisms by which the petal grows into its final anisotropic shape remain to be further understood.

Interestingly, most angiosperm species have specialized conical-shaped cells (conical cells) that are usually found in the petal adaxial epidermis [35,36,37,38]. These cells can vary greatly in size among different species, and conical angles and heights can vary strikingly. As an important feature of the petal epidermis, conical cells are thought to attract pollinators, influence light capture and reflectance, and modulate temperature and wettability [35,36,37,38]; therefore, it is very important to understand how petal cells achieve their characteristic conical shapes. By utilizing new imaging approaches, the Arabidopsis conical cell is becoming a new cellular model system for investigating specialized cell shape [39,40,41]. It has been shown that microtubule reorientation into well-ordered circumferential arrays plays an important role in the determination of the final conical shape [40].

In this review, we summarize microtubule organization during petal morphogenesis. We focus on the role of microtubules in modulating petal growth anisotropy, which has advanced our understanding of how the Arabidopsis petal achieves its characteristic shape. Moreover, we summarize recent live-confocal imaging studies of conical cells. We discuss how microtubule organization is regulated during petal conical cell morphogenesis. Finally, we propose future directions for the study of petal morphogenesis.

## 2. The Arabidopsis Petal

**Figure 1:**
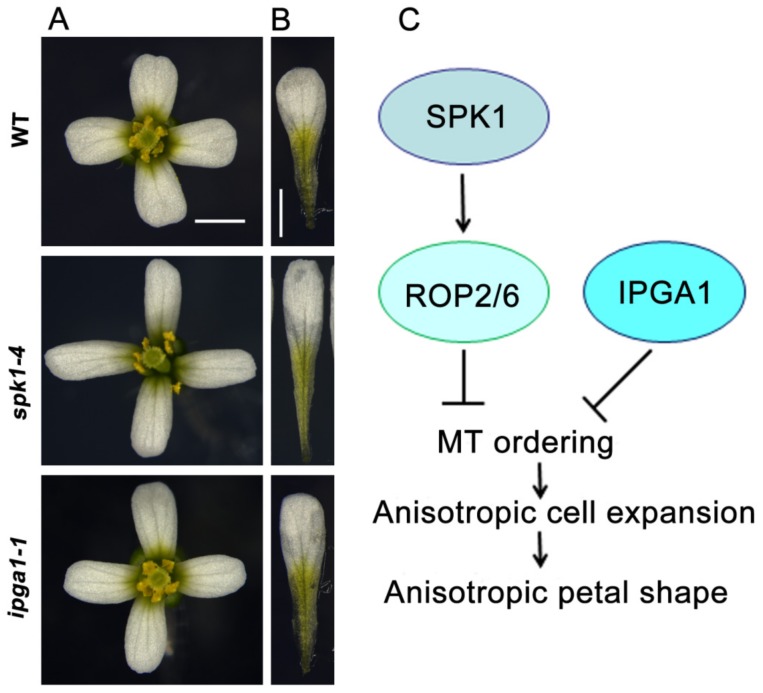
A working model for the regulation of anisotropic petal shape. (**A**) Mature flowers from the wild type, *spk1-4*, and *ipga1-1*. (**B**) Mature petals from the wild type, *spk1-4*, and *ipga1-1*. Bars = 1 mm. (**C**) A working model for anisotropic petal shape control. The SPK1-Rho of Plant (ROP) GTPases-microtubule (MT) signaling module and IPGA1-MT module function in petal anisotropic shape, respectively. SPK1 activates ROP2 and ROP6 to inhibit microtubule ordering, resulting in cell anisotropy and petal anisotropy. IPGA1 negatively regulates microtubule ordering, resulting in cell anisotropy and petal anisotropy. The arrows indicate positive regulation, and the perpendicular lines indicate negative regulation.

We observe, smell, or touch flowers and their petals in different ways, but our knowledge about the molecular mechanisms controlling petal growth and development is very limited. In the past decades, studies of the petal of the model plant Arabidopsis have provided significant insights into how this blade-like simple organ, with approximately 1mm width, arises and differentiates, and forms the final shape [16]. The Arabidopsis petal is a non-essential organ, dispensable for growth and reproduction. Each Arabidopsis plant produces more than 100 flowers, with each flower containing four petals (Figure 1A), enabling experimental manipulations to be performed with ease. The mature petal contains a white blade in the distal region and a basal greenish claw (Figure 1B). The adaxial epidermis in the petal blade contains conical cells, whereas the abaxial epidermal cells are relatively flat with small lobes, and the cells in the claw are more elongated [16,42]. Therefore, the advantage of the Arabidopsis petal is that it provides an elegant model system for investigating organogenesis and cell expansion.

The Arabidopsis petal displays a growth pattern that is similar to other lateral organs. Many growth regulatory genes that were first identified in Arabidopsis petal development also have similar roles in regulating growth and development of other lateral organs [16,17]. During the past decade, a large number of genes have been identified that function in establishing various aspects of cell expansion and petal growth and development [21,22,23,24,25,26,27,28,29,30,31,32,33]. For example, Arabidopsis *JAGGED*, which encodes a zinc finger transcription factor expressed in the distal region of the petal, plays a critical role in petal size, growth, and development. [29,31]. Interestingly, Arabidopsis *RHAMNOSE BIOSYNTHESIS 1* (*RHM1*) encodes a UDP-L-rhamnose synthase, and mutations in this gene influence synthesis of the pectic polysaccharide rhamnogalacturonan-I, and result in left-handed helical growth of petal conical cells and produce twisted petals [33]. Late phases of petal growth and shape formation are largely determined by post-mitotic cell expansion [16,18,19]. The AUXIN RESPONSE FACTOR8 (ARF8) interacts with the basic helix–loop–helix (bHLH) transcription factor BIGPETALp (BPEp) to limit post-mitotic cell expansion and petal size in Arabidopsis [21]. However, despite this progress, regulators that play roles in coordinating post-mitotic cell expansion and anisotropic growth of the petal remain not fully understood.

## 3. The Petal Abaxial Epidermis

### 3.1. ROP Signaling and SPK1 Regulate Microtubule Organization

Plant rho-like small GTPases, usually referred to as Rho of plants (ROP), also known as RACs, which belong to a subfamily of the Rho GTPase family, serve as molecular signaling switches that function in various cellular processes, including cell polarity, cytoskeletal organization, cell wall patterning, and cell morphogenesis [43,44,45,46,47,48,49,50,51]. Like Rho family members from fungi and mammalian cells, ROP GTPases play critical roles in the organization of the actin and microtubule cytoskeleton [52]. In Arabidopsis, ROP GTPases regulate root hair development, pollen tube tip growth, and the interdigitated growth of jigsaw-puzzle shaped leaf pavement cells [43,52]. For example, the phytohormone auxin activates two antagonistic ROP GTPase pathways, which are mediated by two different ROP effectors: The RIC proteins (RIC1 and RIC4), have been proposed to pattern the interdigitated growth of pavement cells [48,53], although the detailed molecular mechanisms remain to be further explored. Auxin plays critical roles in almost every aspect of plant growth and development. The auxin-ROP2-RIC4 pathway promotes the accumulation of actin filaments in the lobe regions; whereas the auxin-ROP6-RIC1 pathway promotes well-ordered transverse microtubule arrays in the indentation regions. Overexpression of ROP6 or RIC1 result in highly ordered transverse microtubule arrays that correlate with reduced interdigitated growth of pavement cells. The *ric1* loss-of-function mutant pavement cells display more randomly oriented microtubules and a wider indentation region of pavement cells than the wild type [53]. RIC1, a microtubule-associated protein, physically interacts with the p60 subunit of the microtubule-severing protein katanin (KTN1). RIC1 promotes the KTN1’s microtubule-severing activity and the formation of the transverse microtubule alignment [53].

Like other members of the Ras superfamily of small GTPase, ROP GTPases function as a molecular switch in plants and shuttle between a GTP-bound active form and a GDP-bound inactive form, which depends on its activating protein (ROPGAP) and guanine nucleotide exchange factor (ROPGEF). ROPGEFs facilitate the release of GDP and ROPGAPs can enhance GTP hydrolysis. Once activated by upstream signals, ROP GTPases associate with their effector proteins to relay signals into downstream components [52]. In the Arabidopsis genome, ROPGEF proteins include two types: the single DOCK180 family of ROPGEFs, SPIKE1 (SPK1) [54,55,56,57,58,59], and the plant-specific ROPGEF family members [51,60,61]. Arabidopsis ROPGEF mutants usually show mild phenotypes, suggesting that they probably function redundantly during plant growth and development. By contrast, SPK1 was identified in a forward genetic screen for Arabidopsis mutants with abnormal trichome development. Loss of SPK1 function leads to seedling lethal and severe defects in organ growth and development, cell-cell adhesion, pavement cell shape, and trichome branching [59]. Using in vitro pull-down assays, it was shown that SPK1 can interact with GDP-bound ROP GTPases. SPK1 was shown to physically interact with the suppressor of cAMP receptor (SCAR)/Wiskott–Aldrich syndrome protein-family Verprolin homology protein (WAVE) complex, which play critical roles in activating actin nucleation/branching by the actin-related protein2/3 (Arp2/3) complex. Genetic and biochemical experiments showed that SPK1, ROP2, SCAR/WAVE, and Arp2/3 complexes function to regulate actin nucleation [54,55,56,57,58,59].

Ren et al., 2016, showed that SPK1 functions in the suppression of anisotropic growth of abaxial epidermal cells during late developmental stages and consequently influencing the final petal shape [23]. *SPK1* knockdown mutants displayed longer and narrower epidermal cells and petals (Figure 1A,B). The increased growth anisotropy in petal abaxial epidermal cells is associated with well-ordered microtubule arrays. As a ROPGEF, SPK1 must activate ROP GTPases and relay the developmental signals to downstream targets [54,55,56,57,58,59]. Analyses of petal phenotypes demonstrated that the triple *rop* mutant (*rop2 rop6 ROP4RNAi*) lines have a significant increase in anisotropic shape of the mature petals, with elongated and narrower morphology, which correlates with increased anisotropic cell expansion and microtubule alignment in the petal abaxial epidermis [23]. A SPK1-ROP GTPases-microtubule-dependent signaling module has been proposed that plays an important role in growth anisotropy and the final shape of Arabidopsis petals (Figure 1C). However, the downstream mechanism by which ROP proteins affects microtubule organization and cell wall patterns remains to be further determined. One possibility is that ROP GTPases may interact with their effectors ICR1 and RIC1 [53,62,63].

### 3.2. IPGA1, a Microtubule-Associated Protein, Regulates Petal Anisotropic Shape 

Live-cell imaging and genetic studies over the past decades have demonstrated that the dynamic features of microtubules, including treadmilling, branching, and severing, enable microtubules to self-organize into diverse arrays, and that microtubule-associated proteins play critical roles in the regulation of microtubule dynamics and organization [64,65,66,67,68,69,70,71,72]. Many mutants of Arabidopsis have been identified that display abnormal cell expansion and developmental defects, correlating with changes in microtubule organization [73,74,75,76]. However, the molecular mechanism by which the microtubule organization is regulated have remained not fully understood. It is possible that previously unidentified microtubule-associated proteins also play roles in microtubule organization during organ growth and development. In addition, how microtubule-associated proteins function in regulating petal anisotropic shape is an open question in Arabidopsis.

To identify novel genes required for regulating petal growth and shape, Yang et al., 2019, performed a genetic screen of ethyl methane sulfonate-mutated lines for mutants with abnormal petal anisotropic shape, and demonstrated that *increased petal growth anisotropy 1* (*IPGA1*) loss-of-function mutants had an elongated-petal phenotype (Figure 1A,B), which is associated with an increase in anisotropic cell expansion of the petal abaxial epidermis [77]. Changes in epidermal cell shape correlates with petal anisotropic growth and shape, particularly at the late stages of petal development, when cell division rates are decreased [77].Analysis of the phenotype of petal abaxial epidermal cells in blades showed that the *ipga1-1* cells displayed an increase in length at stage 10 and beyond, and had a decrease in width at stage 9 and beyond, leading to an increase in cell index (the ratio of length to width) from stages 9 to 14. This result suggests that IPGA1 function is required in the late stages of petal development to restrict anisotropic cell expansion. Map-based cloning studies demonstrated that *IPGA1* encodes an uncharacterized protein containing a coiled-coil region that colocalizes with microtubules and can bind to microtubules in vitro, suggesting that IPGA1 may be a novel microtubule-associated protein.

Analysis of microtubule organization showed that abaxial epidermal cells in the microtubule marker line *GFP-Tubulin6* (*GFP-TUA6*) had randomly-oriented microtubule networks and a few transverse microtubules throughout developmental stages 8–14 [77]. By contrast, petal abaxial epidermal cells of the *ipga1-1 GFP-TUA6* mutant displayed disordered microtubule arrays at stage 8, but had increasingly ordered microtubules throughout petal developmental stages 9–14. Notably, mature cells of *ipga1-1 GFP-TUA6* petals displayed highly aligned microtubules compared with those of the *GFP-TUA6* petals. These results suggested that loss of IPGA1 function leads to a transition, in which microtubule reorganization goes from being random to transverse in the late phases of petal development, and that IPGA1 negatively regulates the organization of microtubules into parallel arrays oriented perpendicular to the axis of petal abaxial epidermal cell elongation [77] (Figure 1C). The IPGA1 family is highly conserved among land plants [77]. However, despite this progress, the IPGA1-interacting proteins are unknown, and the molecular and genetic mechanisms by which IPGA1 regulate microtubule organization, cell expansion, and petal shape need to be further determined.

## 4. The Petal Adaxial Epidermis

### 4.1. Petal Conical Cells

**Figure 2:**
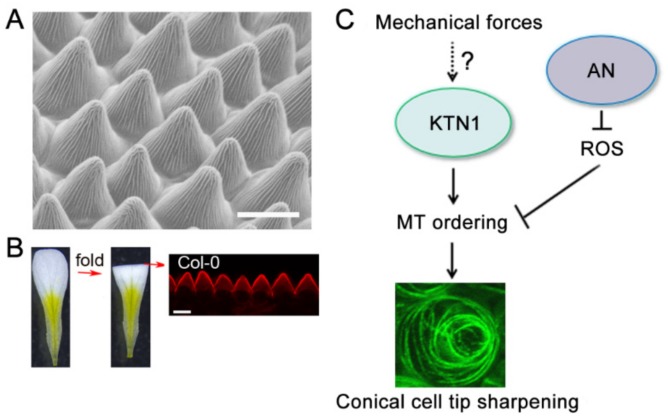
A working model for the regulation of conical cells tip sharpening. (**A**) The petal conical cells were viewed by scanning electron microscopy (SEM). Bar = 15µm. (**B**) The petal conical cells were viewed from a folded petal using confocal microscope. Bar=10 µm. This image was generated from Ren et al. [40]. (**C**) The AN-ROS and KTN1 pathways converge at a node to modulate microtubule ordering during conical cell tip sharpening. AN negatively regulates the level of reactive oxygen species (ROS) and then inhibits the ordering of microtubules, which in turn correlates with the tip sharpening of the conical cell. KTN1-dependent microtubule-severing promotes the formation of well-ordered microtubules and leads to conical cell tip sharpening. Visualization of microtubules in a conical cell from a transgenic line expressing *GFP-TUA6*. Confocal image was generated by top-down 2D maximum projections of Z-stacks. The arrows indicate positive regulation, and the perpendicular lines indicate negative regulation.

Approximately 80% of angiosperm species have specialized conical cells in the petal epidermis [35,36,37,38]. Petal conical cells across various species have been shown to function in pollinator attraction, light capture and reflectance, and maintaining temperature and wettability [35,36,37,38]. The molecular mechanisms that regulate conical cell development remain largely unclear. The R2R3 MYB transcription factor MIXTA in *Antirrhinum majus* has been shown to promote the outgrowth of conical cells from the plane of the petal epidermis, with loss-of-function *mixta* mutants displaying a flat rather than conical shape [36]. Interestingly, this change in petal epidermal cell shape leads to a reduction in the probability that the mutant flowers will be visited by bee pollinators and thereby influences pollination success [36]. Like most angiosperm species, Arabidopsis mature petals have conical cells decorated with cuticular nanoridges in the adaxial epidermis (Figure 2A) [16]. Conical cells may serve as a distinct cell model system for studying complex cell shape in plants.

### 4.2. Live-Confocal and Light Microscopy-Based Imaging of Arabidopsis Conical Cells

The molecular and genetic mechanisms regulating the morphogenesis of conical cells remain poorly understood. Scanning electron microscopy (SEM) is widely used to observe the geometric shape of conical cells at high resolution (Figure 2A), but is not suitable for high-throughput experimental analysis. Live-cell imaging methods, together with powerful software, enable researchers to describe cell morphological changes and gene expression patterns over the course of cell development, and have been widely used in many cell types, such as leaf pavement cells, trichomes, root cells, and shoot apical meristem cells [78,79,80]. Recently, a confocal imaging approach has been developed for investigating conical cell geometry. Petals are transversally folded in half (Figure 2B) [40]. A solution containing propidium iodide was added onto the microscope slide to stain the petals, and which were then observed using a confocal microscope. This allows for a side view of the serrated geometry of conical cells using confocal microscopy (Figure 2B) [40]. In the wild type Arabidopsis, petal adaxial epidermal cells begin to initiate conical outgrowth with a roughly hemispherical morphology at flower developmental stage 8. The cells then undergo both longitudinal elongation and radial expansion after stage 9, with increasing sharpening of the apexes to form a cone over the course of petal development. Notably, a similar method based on light microscopy has been used for the observation of Arabidopsis conical cells [39]. These methods allow for quantitative analyses of the structural parameters (cone angle and height) of the conical cells. Because of the relatively easy and fast sample preparation and image observation, confocal imaging-based high-throughput genetic screening for mutants with conical cell shape defects will be made possible. By using these methods, together with genetic screening, mutants with altered conical cell morphologies have been identified [40,41]. For example, some mutants had an increase in conical cell tip angles compared with the wild type [40,41]. Some mutations specifically influence the dimensions of the epidermal cell cones, with the basal conical region failing to extend to the cell margin, resulting in narrower cones [40]. Another mutant line displayed elongated conical cells [40]. Interestingly, plants carrying mutations in *CYP77A6* were identified displaying cylindrical epidermal cells and had no cuticular nanoridges. It has been proposed that the cuticular nanoridge serves to enhance structural rigidity and help the conical cells suppress conical anisotropic cell expansion to form the cone tips [40].

### 4.3. Microtubule Organization and Conical Cell Expansion

Anisotropic cell expansion is driven by turgor pressure throughout the entire cell surface in plants. The direction of expansion and the final shape are largely determined by the patterning of cell wall architecture through the orientation of cellulose microfibrils [7,8,9,10,11,12]. Cortical microtubules play an important role in guiding the deposition of cellulose microfibrils during cell wall biosynthesis and thus correlate with plant cell expansion. It has been demonstrated that microtubule-associated proteins together with environmental cues, hormone signaling, and mechanical stress play important roles in regulating the dynamics and organization of microtubules [15,81]. However, how microtubules are organized into characteristic arrays during the development of distinct cell types is not fully understood. A recent study using live-cell imaging showed that Arabidopsis wild-type conical cells had randomly oriented microtubules at the early developmental stages, but exhibited increasingly ordered microtubules over the course of cell development [40]. Strikingly, well-ordered circumferentially-oriented microtubule arrays were characteristic features in mature conical cells of the wild type. Surprisingly, by combining genetic and pharmacological experiments using specific inhibitors for microtubules and actin, it was shown that microtubules but not actin filaments play critical roles in conical cell tip sharpening [40].

The microtubule-severing protein KTN1 was originally identified in a screen for mutations corresponding to the mechanical strength of inflorescence stems [82,83]. Loss of KTN1 function caused a severe defect in organ growth and cell shape, correlating with disordered microtubule arrays and abnormal orientation of cellulose microfibrils. Interestingly, *ktn1* mutant mature petals have swollen tips of conical cells with disordered microtubule arrays compared with the wild type [40]. KTN1 is required for microtubule re-orientation from random into well-ordered arrays at late phases of conical cell development; however, the detailed molecular mechanism behind this remains to be further investigated. The KTN1-dependent microtubule-severing activity is at least in part regulated by both RIC1 and SPR2 [53,84]. RIC1, an effector of ROP6 GTPase, activates KTN1 to generate parallel ordering of microtubule arrays in the neck region of leaf pavement cells [85]. SPR2 proteins accumulate at the microtubule crossover sites to prevent microtubule severing by KTN1, which enables randomly oriented microtubule arrays to persist [84]. The KTN1-dependent microtubule organization pattern has been shown to respond to mechanical stress [86], although the detailed mechanism is unknown. Therefore, future studies should aim to investigate the molecular mechanism that controls the spatio-temporal activity of KTN1 over the course of conical cell development.

In a genetic screen for T-DNA insertion mutants with conical cells with wider tip angles, Dang et al., 2018, showed that loss-of-function mutants of *ANGUSTIFOLIA* (*AN*), which encodes a homolog of mammalian CtBP/BARs [87,88], exhibit wider conical cell tip angles [41]. AN plays important roles in modulating cotyledon and leaf shape, cell morphology, and microtubule organization. *AN* loss-of-function mutants have narrower cotyledons and reduced interdigitation of pavement cells with well-ordering microtubule arrays. The swollen conical cell phenotype observed in the *an* mutants is correlated with an increase in accumulation of reactive oxygen species (ROS) in the *an* mutant conical cells [41]. ROS function as signaling molecules in normal cellular processes and organ growth and development in plants [89].

Both exogenously supplied ROS and reduced endogenous ROS levels can generate similar conical cell phenotypes resembling that of the *an* mutants, suggesting that ROS homeostasis plays critical roles in modulating conical cell tip sharpening [41]. Interestingly, it was shown that AN interacts with several proteins responsible for ROS homeostasis. Therefore, these results suggest that ROS may act downstream of AN in the control of conical cell shaping (Figure 2C). Previous studies have shown that ROS play critical roles in modulating cytoskeleton dynamics [90,91,92], and that H_2_O_2_ can directly activate the mitogen-activated protein kinase (MAPK) cascade to influence the activity of MAP65 [90], which in turn affects microtubule organization. ROS play an important role in microtubule organization in conical cells [41]. Both exogenously supplied H_2_O_2_ and eliminating endogenous ROS can result in alterations in microtubule orientation in conical cells, suggesting that ROS homeostasis plays critical roles in mediating microtubule re-orientation into well-ordered circumferential arrays in conical cells, although the underlying mechanism remains to be further investigated. Consistent with the role of AN in negatively regulating ROS production, AN was shown to positively regulate microtubule ordering in conical cells. Interestingly, genetic analyses have demonstrated that AN and KTN1 act in parallel pathways to modulate conical cell tip sharpening [41]. Therefore, it is possible that the AN-ROS and KTN1 pathways converge at a node to modulate microtubule ordering during conical cell tip sharpening (Figure 2C).

## 5. Conclusions and Perspectives

While the concepts of cell morphogenesis and organ shape are increasingly well characterized in leaves, the mechanism of shape formation in petals remains poorly understood. Arabidopsis petals have long been recognized as an ideal model system for the study of anisotropic shape formation and plant organogenesis, and recent studies have proposed that petal epidermal cells serve as a valuable model system for studying how specific epidermal cell types reproducibly develop into their characteristic shapes [16,17,23,40,41]. Regulation of microtubule organization and dynamics plays an important role in cell expansion and shape and plant morphogenesis [64,65,66,67,68,69,70,71,72]. Microtubules guide the orientation of the cellulose microfibrils and pattern of the cell wall. Expanding plant cells are restricted by the primary cell wall, which contains cellulose microfibrils embedded in a matrix of pectic polysaccharides and hemicellulose. In Arabidopsis cotyledons and leaves, pavement cell shaping is thought to link microtubule organization and mechanical stress [86,93,94]. A long-standing view is that microtubule arrangement is usually aligned along the main mechanical stress direction. Microtubules in turn pattern the biosynthesis of cellulose microfibrils, and thus generate cell wall reinforcement, which provides a mechanical-dependent feedback loop for the regulation of pavement cell shaping [86]. Interestingly, a recent study showed that the shape of jigsaw-puzzle cells with lobes is an adaptation adopted by various plant organs to resist the mechanical stress [95].

In contrast to the extensive studies over the past several decades conducted to further our understanding of the mechanisms that pattern the morphogenesis of diverse cell types [96,97,98,99,100,101,102], such as pavement cells, root hairs, and conical mesophyll cells, the mechanisms that control the outgrowth and the anisotropic expansion of petal conical cells remain largely unknown. Many biomechanical concepts have been proposed to illustrate lobe formation of pavement cells in leaves [92,93,95]. Future studies should aim to investigate whether and how conical cells generate mechanical stress. Auxin functions as a key regulator during plant growth and development. In leaf pavement cells, polarized subcellular distributions of the auxin efflux carrier PIN1 that generate subcellular auxin gradients correlate with lobe initiation of pavement cells [48]. However, this model has been challenged by a recent study showing that PIN proteins were not correlated with lobe patterns of pavement cells [103]. Although PIN proteins have been shown to localize to plasma membrane in the petal cells [29], whether and how auxin triggers cell expansion and shape expansion in Arabidopsis petals remains poorly understood. Further studies should investigate the roles of hormone signaling, mechanical forces, and cell wall patterns during petal development and morphogenesis and conical cell shaping. It is possible that cell wall matrix polysaccharide distribution may play a critical role during petal cell growth.
